# Objective assessment of mental stress in individuals with different levels of effort reward imbalance or overcommitment using heart rate variability: a systematic review

**DOI:** 10.1186/s13643-022-01925-4

**Published:** 2022-03-19

**Authors:** Beatrice Thielmann, Jonas Hartung, Irina Böckelmann

**Affiliations:** Institute of Occupational Medicine, Medical Faculty, Otto-von-Guericke-University, Magdeburg, Leipziger Str. 44, (Building 20), 39120 Magdeburg, Germany

**Keywords:** Heart rate variability, Workload, Mental stress, Employees, Holter ECG

## Abstract

**Background:**

Workloads are increasing and could cause mental stress, e.g., in the form of subjective effort reward imbalance (ERI) and overcommitment (OC). The heart rate variability (HRV) is a valid method for objective monitoring of workload. The aim of this project is to systematically evaluate the literature on HRV as an objective indicator for mental stress in individuals with different levels of ERI or OC.

**Methods:**

A systematic literature review examining HRV of employees in accordance with the Preferred Reporting Items for Systematic Reviews and Meta-Analysis (PRISMA) statement for reporting systematic reviews was performed. Electronic databases used were PubMed, Ovid, Cochrane Libary, Scopus and Web of Science, PsyInfo, Psyndex, and Livio. Only articles from 2005 to 2021 were included. Inclusion criteria were case-control studies, intervention studies, cross-sectional studies, or longitudinal studies with different levels of ERI and/or OC, >10 participants in each group, measurement of 24h HRV by using Holter ECG or chest belt, and full-text in English or German language. The methodological quality was evaluated by using a modified STARD for HRV.

**Results:**

Five studies matched the inclusion criteria by using HRV (24-h ECG) with a different HRV analysis at day and night. It showed an adaptation of HRV with higher ERI or OC with reduced parasympathetic HRV parameters, but the studies were not comparable.

**Conclusions:**

There is a need for occupational health studies that examine strains and stress of different employees with predominantly mental stress. The well-established parasympathetic mediated HRV parameters seem to be suitable parameters to objectify the stress.

**Supplementary Information:**

The online version contains supplementary material available at 10.1186/s13643-022-01925-4.

## Background

No matter whether it is an obligation or vocation, work remains a central topic for every individual. In this context, we are facing a working society in a state of ongoing change. The world of work is becoming more diverse, more digital, and more global. It provides new opportunities, but also risks. Currently, four generations are working together in many branches [1]. From baby boomers to Generation X and Y to Generation Z, which could not be more different. They have different claims on work and leisure time. This also results in different ideas of loyalty and flexibility [1]. As a result, stress in the workplace can be perceived differently and making occupational health assessments necessary on an ongoing basis and requiring constant reassessment.

An established subjective assessment instrument for mental stress is the effort reward imbalance model (ERI) according to Siegrist [2]. The ERI questionnaire reveals satisfactory psychometric properties and can be recommended for further research in the era of economic globalization [3]. The model is used to determine the relationship between the work performance/overcommitment (effort) and the experienced reward [2]. The baseline assumption of the model is that an imbalance between the lack of occupational rewards and the expenditures can lead to adverse stress reactions. If the reward perceived after work performance becomes insufficient, a specific form of social crisis may occur—the so-called gratification crises [2]. Here, individually and socially expected relationships are disappointed. The concept of ERI is exposed to enormous subjective individual variations in a defined work environment and is evaluated very differently between individuals [2]. In this regard, ERI values below 1.0 indicate a balance between effort and reward; values above 1.0 indicate an imbalance of effort and reward [2, 3]. Various studies have shown, for example, an increased risk of cardiovascular disease [2, 4] and the increased occurrence of psychological symptoms such as depression [5, 6] in association with a high ERI ratio.

The overcommitment (OC) subscale of the ERI describes the tendency to overspend oneself without regard to one’s resources [2]. So it is an intrinsic, person-related factor. Overcommitment is also associated with health risks. It is associated with vital exhaustion [7] or burnout [8]. Furthermore, it can lead to musculo-skeletal disorders [9], inflammation [10], or impaired immunocompetence [10].

Heart rate variability (HRV) analysis is a possible method for objective monitoring of workload, e.g., in the context of an occupational health examination [11]. Guidelines define HRV as variations over time between consecutive heartbeats. They also see HRV as a very sensitive indicator of dysregulation of the autonomic nervous system (ANS) [12, 13]. It is a non-invasive measurement to evaluate the stress of the cardiovascular system [14]. The vagus nerve, which stimulates the atria of the heart and modulates the self-sustaining sinus rhythm of the sinus or Keith flack node, is an essential part of HRV tone. The interaction between sympathetic and parasympathetic nervous systems can be estimated as different demands with the analysis of HRV [13]. Parasympathetic activity dominates in rest and recovery phases of the body, whereas sympathetic activity dominates in chronic state of stress [13]. HRV analysis differs time, frequency, and nonlinear domains. An overview of HRV metric is given by [14, 15], or the current guidelines [12, 13]. The ANS is involved in stress regulation, so (work-related) chronic stress has been associated with reduced HRV and reduced parasympathetic modulation [16]. For example, HRV markers of vagal function are the root mean square of successive differences (RMSSD), percentage of successive NN intervals that differ by more than 50 ms (pNN50), high frequency power (HF), and standard deviation of point plot to the transverse diameter (SD1) [13]. But other parameters (e.g. low frequency power (LF), LF/HF ratio (LF/HF)) are without clear assignment and can be influenced by the sympathetic and parasympathetic nervous system [13]. Analyzing HRV, it should be noted that there is an age dependency of HRV [17], and it is also necessary to know which recording time is necessary (e.g., 24-h, short-term (5 min), and ultrashort-time (<5 min)) for according parameters and which parameters are relevant for the question to be determined [18].

The aim of this project was to systematically evaluate the literature on heart rate variability as an objective indicator for mental stress in individuals with different levels of ERI and/or OC. We hypothesized that a high ERI ratio or high OC is associated with an increased reduction in vagal tone.

## Methods

This systematic literature review examined heart rate variability in context of effort reward imbalance and/or overcommitment in accordance with the Preferred Reporting Items for Systematic Reviews and Meta-Analysis (PRISMA) statement for reporting systematic reviews [19]. The electronic databases PubMed, Ovid, Cochrane Library, Scopus and Web of Science, PsyInfo, Psyndex, and Livio were used. The deadline was February 01, 2021. Search terms were defined as “overcommitment” OR “effort reward imbalance” AND “heart rate variability” OR “HRV” OR “cardiac autonomic control” OR “autonomic function” OR “parasympathetic activity” OR “parasympathetic nervous system” OR “cardiac vagal tone” OR “autonomic cardiac modulation” OR “vagus nerve” OR “vagal tone” OR “vagal activity” OR “coefficient of variation” OR “autonomic nervous system OR “sympathetic” OR “parasympathetic” OR “sympathetic nerve activity” OR “neural control” OR “activation of the sympathetic nervous system”. Only articles from 2005 to 2021 were included. Inclusion criteria were studies with different levels of ERI and/or OC, more than 10 participants (in each group), measurement of HRV 24 h, recording of heart rate through Holter ECG or chest belt, full-text in English or German language, and human subjects. Papers with case-control studies, intervention studies, cross-sectional studies, or longitudinal studies were included.

Exclusion criteria were HRV assessment with pulse rate automatic or photoplethysmography, diagnosis of mental or neurological diseases, endocrine diseases (diabetes, thyroid gland disease), cardiac diseases, hypertension, other heart rhythm-related diseases, and intake of drugs influencing HRV. Review articles, guidelines, single-case studies, theses, dissertations, and scientific conference abstracts were also excluded. The national guideline on HRV does not suggest the method of pulse rate or photoplethysmography of measurement [14], so that was an exclusion criteria.

**Fig. 1:**
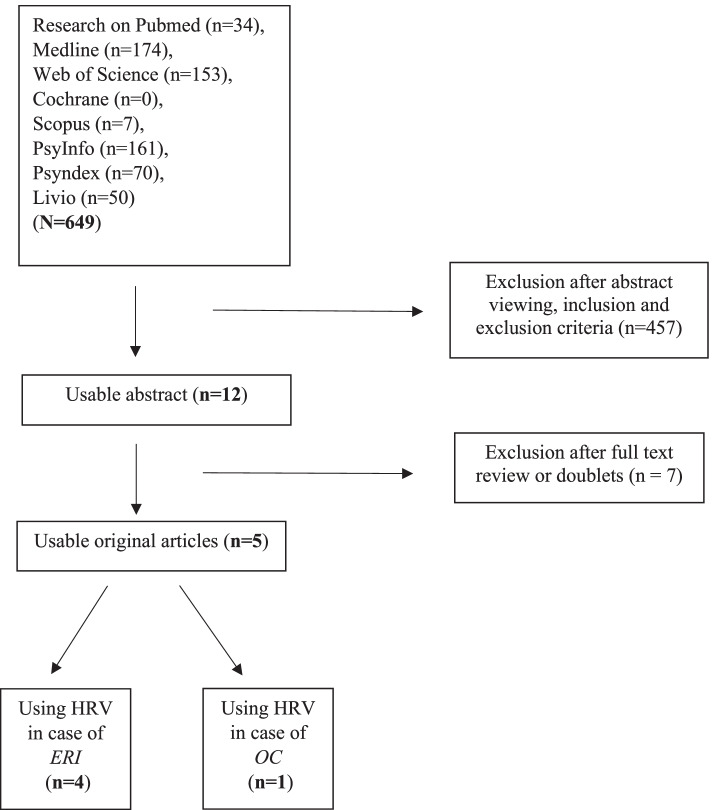
Flow chart in the context of the systematic literature search

Next to the literature research, a hand search was performed by checking the reference lists of the included studies (no result). One study was included in the databases after the literature search (due to a subsequent publication). An overview of the procedure is shown in Fig. 1. The complete study protocol is available at Prospero https://www.crd.york.ac.uk/PROSPERO/display_record.php?RecordID=234228.

The included articles were transferred to the reference manager Citavi 6 (Swiss Academic Software, Wädenswil, Switzerland) without duplicates. Two authors (B.T. and J.H.) independently screened titles and abstracts according to the inclusion and exclusion criteria. The full-text of each relevant article was obtained, which was independently screened by two authors (B.T. and J.H.). If no full-text was available, the authors were contacted. Disagreements were resolved through discussion with a third reviewer (I.B.).

The methodological quality of the research question relevant studies was evaluated using the Standard for Reporting Diagnostic Accuracy Studies (STARD) guidelines [20, 21], which follows the recommendations of [22] and [18]. All studies were also evaluated independently by two authors (B.T. and J. H.) using a modified STARD for HRV by [23]. It included 25 items (maximum of points). We have slightly modified two assessment tools [24], but the maximum score did not change. The items are shown in Table 1. Disagreement was solved by (I.B.) and discussion.

**Table 1:** Evaluation points (P) of STARDHRV followed by Dobbs et al. [23] and modified by Grässler et al. [24]

		Evaluation point	Points	Assessment standard
1	Title or Abstract	Identification as a study of validation	1	Reported
			0	Not reported
2	Abstract	Structured summary of study objective. Design. Methods. Results. Conclusions	1	Yes
			0.5	Yes. But not structured
			0	Not reported.
3	Introduction	Scientific and practical background. including the intended use of the index device/software	1	Complete. Including the application of the HRV method
			0.5	With limitations available
			0	Insufficient background.
4		Study objectives and hypotheses described	1	Study objectives and hypothesis
			0.5	Study objectives without hypothesis
			0	Not reported
5	Methods	Study uses within-subject design	1	Reported
			0	Not reported
6		Intended sample size and how it was determined (e.g., G*Power 3)	1	Reported
			0	Not reported
7		Eligibility criteria including specific restrictions (medical use, gender, age, activity level or body mass index (BMI))	1	Reported for health, medical use, gender, age, activity, and BMI
			0.5	Reported in some criteria less than 1P
			0	Not reported
8		Pre-testing guidelines reported (e.g., limitations to caffeine, alcohol, and physical activity)	1	Reported for limitations to caffeine, alcohol, and physical activity
			0.5	Reported in some criteria less than 1P
			0	Not reported
9		Setup of reference standard and index device described in sufficient detail to allow replication (e.g., hardware/software such as brand and electrode configuration)	1	Sufficient description. A replication is possible.
			0.5	Limited description. A replication is partially possible
			0	Insufficient description. Replication is not possible
10		Description of environmental conditions (e.g., temperature, humidity, lights on or off, and time of day) and posture	1	Temperature + time of day or same time of day + body position
			0.5	Reported in some criteria less than 1P
			0	Not reported
11		A stabilization period prior to sampling was described	1	Yes. With information about when and how long
			0.5	Yes. With information about when or how long
			0	Not reported
12		The raw sampling rate and length of collection are described	1	Sampling rate + length of collection
			0.5	Only length of collection
			0	Not reported
13		Acknowledgment of breathing (e.g., controlled or not controlled)	1	Reported
			0	Not reported
14		Description of how estimates or comparison measures were calculated (e.g., ES, LOA, Pearson’s *r*, or ICC)	1	Reported
			0	Not reported
15		Reasons for missing data. along with percentage missing (e.g., equipment. persistent ectopy) and how it was handled	1	Reasons for missing data + percentage + handling
			0.5	Reported in some criteria less than 1P.
			0	Not reported
16		Interbeat artifact identification method (e.g., algorithm and manual inspection)	1	Manual inspection of artifacts
			0.5	Automatically without manual correction
			0	Not reported.
17		Artifact cleaning methods and percentage of beats corrected	1	Description method (e.g., smoothing or decimation) and percentage
			0.5	Reported in some criteria less than 1P
			0	Not reported
18		Description of metrics used and software/script for HRV calculation (log transformation)	1	Reported
			0	Not reported
19		Specification of frequency bands used and how they were calculated (e.g., fast Fourier transform (FFT) or autoregressive modeling (AR))	1	Reported
			0	Not reported
20	Results	Baseline demographics of participants	1	Reported
			0	Not reported.
21		Mean ± SD along with at least one estimate of precision (e.g., LOA, Pearson’s *r*, or ICC)	1	*p* values and effect size
			0.5	Only *p* values
			0	Not reported
22	Discussion	Study limitations (e.g., sources of potential bias, confounding variables, statistical uncertainty, and generalizability)	1	In detail (if necessary also as extra section)
			0.5	Discussed, but not in detail
			0	Not reported
23		Implications for practice including the intended use	1	Detailed, giving practical recommendations (e.g., clientele and how often), extra section
			0.5	Discussed, but not in detail
			0	No statement or simple statement “We have seen differences and suggest that”
24	Other information	Where the full study protocol can be accessed if not fully described	1	Reported
			0	Not reported
25		Sources of funding and other support; role of funders	1	Information about funding and conflict of interest
			0.5	Funding. conflict of Interest or acknowledgement
			0	Not reported

From the included studies, the changes in all HRV parameters used were collected. Due to the limited data available, a descriptive discussion of the results was conducted without further statistical analysis. Increases were marked with an upward arrow, decreases with a downward arrow, and no change with an arrow pointing to the left and right. Significant changes were marked with an asterisk. Table 2 explains the parameters used in the review and the affiliation to the ANS.

**Table 2:** Overview of the HRV parameters evaluated in the review and their importance

HRV parameter	Definition and explanation	Activity as part of the autonomic nervous system
**Time domain**		
SDNN [ms]	Standard deviation of all normal-to-normal R-R (NN) intervals	Sympathetic and parasympathetic nervous system
SDINN Index	Mean of the 5-min standard deviation of the NN interval	No clear assignment
SDANN [ms]	Standard deviation of the average of NN intervals in 5-min segments	No clear assignment
RMSSD [ms]	Root mean square of successive differences of NN intervals	Parasympathetic nervous system
pNN50 [%]	Percentage of successive NN intervals that differ by more than 50ms	Parasympathetic nervous system
**Frequency domain**		
HF [ms^2^]	High frequency power	Parasympathetic nervous system
LF [ms^2^]	Low frequency power	Sympathetic and parasympathetic nervous system
LF/HF	LF/HF ratio	Quotient between LF and HF power

## Results

The initial search resulted in 649 records and included one study, which was published after literature research [25]. After removing duplicates and exclusions based on title and abstract, only five full-texts were assessed for eligibility. Four studies used ERI [26–29], and one study used OC [25]. The professional groups were different (four studies): nurses [26, 27], employees of different sectors/branches [28, 29], and kindergarten teachers [25]. Two studies studied the same subjects, but reported different HRV parameters in the two publications, so they were both listed [26, 27]. All studies came from Europe (Germany and Italy). An overview of the included studies is shown in Table 3. The literature search revealed five studies with HRV analysis using ERI and/or OC, but ECG recordings were too short (3 min, 45 min, 2 h, 18 h) or too long (36 h), so they were excluded from the review [30–34]. Only one study examined risk factors related to cardiovascular disease, but only with the glycemic status [28]. All studies examined daytime and nighttime separately. Subject populations varied widely, and ranged from 53 [26, 27] to 9937 [28]. All study protocols were different. Two studies used classification with the ERI ratio [26, 27] and one a cutoff of OC [25], and each compared the groups. One study divided into age groups, compared them with RMSSD as the only parameter, and included ERI as a coefficient [29]. One study averaged ERI and RMSSD and ran various model calculations. Glycemic status and the inflammation parameter CRP were also included [28]. One study examined only women [25], two with more than two-thirds [26, 27], and two with less than 20% [16, 29]. Where possible, no gender differences were found in the studies.

**Table 3:** Results of the systematic research

Author, year	Country, profession	Characteristics of subjects	Method		Outcome and measurement of HRV	STARD HRV
			ERI/OC	HRV		
Using ERI						
Borchini, 2015 [26]	Italy, nurses	*n*=53 SLS *n*=36, RHS *n*=7, PHS *n*=10 Women: SLS 79%, RHS 86%, PHS 90% Age: SLS 37.2±1.9 years, RHS 40.3±2.8 years, PHS 41.0±4.1 years Healthy	SLS=ERI 0.5±0.2 RHS=ERI 1.1±0.4 PHS=ERI 1.7±0.6	24-h ECG Holter Between WD, RD Day, night	*WD:* SDNN: SLS>RHS>PHS*; SDNN_Index: SLS>RHS>PHS*; SDANN: SLS>RHS>PHS; RMSSD: SLS>RHS>PHS; pNN50: SLS>RHS>PHS. *RD:* SDNN: SLS>RHS>PHS; SDNN_Index: SLS>RHS>PHS; SDANN: SLS>RHS>PHS; RMSSD: RHS>SLS>PHS; pNN50: SLS>RHS>PHS	16
Borchini, 2018 [27]	Italy, nurses				*WD_working*: HF: SLS>RHS>PHS*; LF: SLS>RHS>PHS*, LF/HF: RHS>SLS>PHS. *WD_non-working:* HF: SLS>RHS>PHS; LF: SLS>RHS>PHS*; LF/HF: SLS=RHS>PHS. *WD_night: HF:* SLS>RHS>PHS; LF: SLS>PHS>RHS; LF/HF: PHS>SLS>RHS. *RD_day:* HF: SLS>RHS>PHS; LF: SLS>RHS>PHS*, LF/HF: SLS>RHS>PHS. *RD_night:* HF: SLS>RHS>PHS; LF: SLS>RHS>PHS; LF/HF: SLS=PHS>RHS	19.5
Jarczok, 2016 [28]	Germany, employees (sec./tert. sector)	*n* = 9.937 (women: 18.2%) Age 41.2 ±10.6 years Not reported for diseases, medication, online questionnaire	ERI 1.2±0.5 Distinct mediation models Structural equation	24-h ECG Chest belt Between	ERI neg. associated RMSSD (↓) at day and night	15
Loerbroks, 2010 [29]	Germany, employees (airplane manufacturer)	*N*=581 (women 11.5%) AG I *n*=159, AG II *n*=158, AG III *n*=183, AG IV *n*=81 Age range [years]: AGI I 17–34, II 35–44, III 45–54, IV 55–65	Age groups ERI as coefficient	24-h ECG Holter Between	ERI neg. associated with RMSSD (↓) over WD, not during sleep Most pronounced in workers aged 35–44 years Effect of age	17
Using OC						
Darius, 2021 [25]	Germany, kindergarten teachers	*n* = 163 (100% women) Age 45.5±12.4 years healthy	OC high/low Cut off ≥ 18	24-h ECG (Holter) 24 h, 6 h day, 6 h night Between	24h: OC high: ↓ RMSSD*, pNN50*, SDNN, HF; ↑ LF, LF/HF Day: OC high: ↓ pNN50, SDNN, HF; ↔ RMSSD, LF/HF, ↑ LF Night: OC high: ↓ RMSSD*, pNN50*, SDNN*, HF; ↑ LF, LF/HF Effect of age: LF, HF in night, not effect of work experience	16

*HRV parameter* time domain—SDNN (standard deviation of all normal-to-normal R-R intervals), *SDNN_ Index* (mean of the 5-min standard deviation of the NN interval), *SDANN* (standard deviation of the average of NN intervals in 5-min segments), *RMSSD* (root mean square of successive differences of R-R intervals, *pNN50* (percentage of successive NN intervals that differ by more than 50 ms); frequency domain—LF (low frequency power, 0.04–0.15 Hz), *HF* (high frequency power, 0.15–0.4 Hz), LF/HF-ratio

*Between* between-subject design, *ERI* effort reward imbalance ratio, *OC* overcommitment. Significant *p* values are marked with asterisks (* for *p*<0.05). *SLS* stable low strain, *RHS* recently high strain, *PHS* prolonged high strain, *WD* working day, *RD* resting day, *AG* age groups

### Outcome heart rate variability

One study used a chest belt [28], and the other four used classic Holter ECGs.

The time periods for HRV analysis varied widely among the studies. Borchini et al. analyzed 2 h of the 24 h recordings, each at the 5 different phases (working day working, non-working, night and resting day with day and night phase) [27]. The duration of HRV derivation in each phase was not standardized. Table 3 presents the outcome of all HRV measures.

Four studies used RMSSD as a marker of vagal function [25, 26, 28, 29]. RMSSD decreased with higher ERI or OC outcomes. It was significant for 24 h and night phase [25], overworking day, but not sleep [29], and also negative associated with ERI [28]. No significance was found in one publication [26]. The parasympathetic-associated parameter pNN50 decreased in kindergarten teachers with high overcommitment in 24 h and night phase [25]. For the SDNN (parasympathetic and sympathetic nervous system), SDANN and SDNN Index (both parameters without clear assignment to parasympathetic or sympathetic nervous system) at working day [26] and for SDNN in night phase [25] are decreased in subjects with higher ERI or OC. The frequency domain parameter HF showed the same tendency [25, 27]. The two studies that used LF and LF/HF showed opposite trends. LF and LF/HF increased at higher ERI [25], but also decreased [27]. The trend of HRV parameters looks adaptive to the stress situation related to higher ERI or OC.

One study found age-dependent effects for LF and HF at night. This study also examined work experience, which had no effect on HRV [25]. The study with age-related research found a lower RMSSD in higher ERI, which was most pronounced in employees aged 35–44 years [29].

### Quality assessment

The study quality of HRV methodology was evaluated with STARD_HRV_ [23] and modified according to [24]. The scores for all studies were 15 [28], 16 [25, 26], 17 [29], and 19.5 [27].

Full marks were achieved in all studies for points 1, 2, 9, 14, and 29. Zero points were found in the case of elevation points 5, 6, and 13 in all studies. The other points showed a heterogeneous allocation from 0 to 1. This evaluation is attached as Supplement 1.

Monitoring during the work could lead to movement artifacts, which limits the assessment. Three studies reported exclusion criteria about diseases and medication [25–27] and two did not report [28, 29]. Three studies performed a manual inspection of NN intervals [25–27]; other publications did not do so [28, 29]. Only one of the studies reported the percentages of adjusted material [29].

### Summary of the results

The observed studies showed an adjustment of HRV by reduction of parasympathetic mediated HRV parameters thus at higher subjective stress (higher ERI or OC). The study quality of the HRV methodology was moderate. The average score for all studies was 16.7/25 points.

## Discussion

The purpose of the review is to systematically evaluate the literature on heart rate variability as an objective indicator for mental stress in individuals with different levels of ERI and/or OC.

All studies used HRV during work and examined day and night phases. The selected HRV parameters are able to provide information about the measured strain (effort reward imbalance and/or OC). It should be noted that there are different study protocols and different recording times, so these values are only comparable to a limited degree.

Comparisons and statements about cardiovascular risk factors cannot be made. No gender differences were found on the basis of the studies either.

Deficiencies were found in the methodological quality and in the quality of the study reports. The numbers of subjects are very small (except for one study), so a generalization is not possible.

A trend can be seen so that the predominantly parasympathetic mediated parameters (e.g., RMSSD, pNN50, HF) decreased as an adaptation to workload (high ERI or OC) with a decrease. HRV parameters with both parasympathetic and sympathetic influences also decreased (e.g., SDNN, SDANN) or increased (e.g., LF, LF/HF). This is concerning, especially if HRV cannot be adequately adjusted by nighttime sleep, which hypothesizes a lack of recovery. Nonlinear parameters were not used. Minor age-related effects and not effects of work experience of HRV parameters could be found; both should not be overinterpreted.

## Conclusions

This systematic review shows that there is a high need and a great potential for occupational health studies among different professional groups with mental stress. HRV is a valid objective method for visualizing stress, i.e., for measuring strain [13]. We recommend the use of 24-h ECGs to evaluate the “night” recovery phase. For the assessment of mental stress, the parasympathetic dominant HRV parameters were shown to be effective markers for this. Other parameters (e.g., without clear assignment or nonlinear parameters) should be used as a complement.

## Supplementary Information

**Additional file 1: Supplement 1**. Results of the STARD_HRV_ evaluation of included publications.

## Data Availability

The data can be accessed via the corresponding author. They are archived at the corresponding university.

## Abbreviations

AG: Age groups
ANS: Autonomic nervous system
ERI: Effort reward imbalance ratio
HF: High frequency power
HR: Heart rate
HRV: Heart rate variability
LF: Low frequency power
LF/HF: Quotient between LF and HF power
OC: Overcommitment
pNN50: Percentage of successive NN intervals that differ by more than 50ms
PHS: Prolonged high strain
RD: Resting day
RHS: Recently high strain
SDANN: Standard deviation of the average of NN intervals in 5-min segments
SDNN: Standard deviation of all normal-to-normal R-R intervals
SDNN Index: Mean of the 5-min standard deviation of the NN interval
SLS: Stable low strain
WD: Working day
